# Association between risk-related lifestyle factors, screening adherence and breast cancer incidence: a decomposition analysis

**DOI:** 10.1038/s41598-026-58989-8

**Published:** 2026-06-22

**Authors:** Md Sohel Rana, M. Luke Marinovich, Nehmat Houssami, Dominic Cavenagh, Julie E. Byles, Xue Qin Yu, Md Mijanur Rahman

**Affiliations:** 1 The Daffodil Centre, The University of Sydney and Cancer Council NSW, Sydney, Australia; 2 Department of Statistics, Comilla University, Kotbari, Cumilla, 3506 Bangladesh; 3 Sydney School of Public Health, Faculty of Medicine and Health, The University of Sydney, Camperdown, Australia; 4 Centre for Women’s Health Research, University of Newcastle, Newcastle, Australia; 5 Hunter Medical Research Institute, New Lambton Heights, Australia; 6 University of Newcastle, Callaghan, Australia; 7 The Collaboration for Cancer Outcomes, Research and Evaluation (CCORE), Ingham Institute for Applied Medical Research, Liverpool, NSW Australia; 8 School of Clinical Medicine, South-Western Sydney Clinical Campus, University of New South Wales, Sydney, Australia

**Keywords:** Lifestyle factors, Breast cancer, Screening adherence, Four-way decomposition, Cancer, Diseases, Health care, Medical research, Oncology, Risk factors

## Abstract

Adherence to breast cancer (BC) screening may have mediated the association between lifestyle-related risk factors and BC incidence, but evidence on this hypothesis remains limited. This study aimed to decompose the overall effects of lifestyle factors on BC incidence, considering the mediating effect of screening adherence. In this longitudinal cohort study with follow-up from 2001 (cohort age: 50–55) to 2019 (cohort age: 68–73), we used a four-way decomposition approach to break down the overall effect of lifestyle factors on BC incidence into direct effects and mediated effects through screening adherence to quantify their total excess risk ratios (TERRs). Of the 10,579 women included in the study, 5.40% (*n* = 571) were diagnosed with BC during follow-up, with a higher proportion among those who adhered to BC screening (5.83%, *n* = 474) than non-adherers (3.96%, *n* = 97). The overall increased risk of BC was observed for higher body mass index (BMI) (TERR = 1.30), alcohol consumption (TERR = 1.55), and hormone replacement therapy (HRT) use (TERR = 1.57) (all *P* < 0.0001). The direct effects remained significant for BMI (excess relative risk [ERR] = 0.37, *p* = 0.001), alcohol consumption (ERR = 0.44, *p* = 0.003), and oral contraceptive use (ERR = − 0.69, *p* < 0.001) after adjusting for the mediation effect of screening. Significant indirect effects were also observed for alcohol consumption (3.32%, *p* = 0.001) and HRT use (5.08%, *p* < 0.001). These findings underscore the complex nature of BC risk and highlight the importance of considering both lifestyle factors and screening in cancer prevention strategies.

**Supplementary Information:**

The online version contains supplementary material available at https://doi.org/10.1038/s41598-026-58989-8.

## Introduction

Breast cancer (BC) is the most frequently diagnosed cancer among women, with an estimated 2.3 million new cases in 2022 and remains the leading cause of death^1^. Early detection through regular mammography screening can improve BC outcomes by optimising treatment benefits^2^. Despite the well-documented benefits, uptake of BC screening is not consistent across populations. The frequency of mammography screening for women can vary depending on several lifestyle, sociodemographic, reproductive and psychological factors^3^.

The associations between lifestyle factors such as alcohol consumption, physical activity, body mass index (BMI), smoking, diet, breastfeeding, oral contraceptive (OC) use, and hormone replacement therapy (HRT) use, and BC incidence are well documented^4–10^. However, the pathways underlying such relationships remain largely unclear, especially around whether adherence to mammography screening mediates the association. Screening adherence is likely to be correlated with lifestyle factors; for example, the likelihood of adherence to screening among overweight/obese women is lower compared to those of standard weight^11–13^, which ultimately delays diagnosis. On the other hand, those who are health-conscious (for example, moderate or non-alcohol drinkers or non-smokers) may be more likely to participate in regular screening^12,14,15^. Understanding the direct and indirect effects of lifestyle factors on BC incidence in the presence of screening adherence as a mediator is relevant for precise risk classification and designing intervention programs. However, screening adherence and the influence of lifestyle factors on BC incidence have been less studied.

Typical regression models may be insufficient to disentangle the direct and indirect effects of lifestyle factors on BC incidence, where there may be indirect effects mediated by screening adherence. Mediation analysis, such as the four-way decomposition approach, is a rigorous structure for dealing with such complexity^16^. The four-way decomposition method permits partitioning the total effects of an exposure on an outcome in the presence of a mediator into four distinct components: (1) controlled direct effect (unmediated by screening), (2) pure indirect effect (mediated solely through screening), (3) reference interaction (screening adherence-independent effect modification), and (4) mediated interaction (screening adherence-dependent effect modification). Understanding these decomposed effects may be valuable in shaping efficient public health policies.

There is a lack of evidence regarding the mediating role of screening adherence in the pathway of association between lifestyle factors and BC incidence, which rather investigates lifestyle factors in isolation or treating screening adherence as a confounder^17^. This study aimed to investigate the influence of risk-related lifestyle factors on BC incidence in the presence of screening adherence as a mediator. By evaluating these pathways, we provide potentially valuable mechanistic insights to inform targeted interventions that promote healthy lifestyles and BC screening.

## Results

Of 10,579 participants, just over three-quarters (*n* = 8130, 77%) adhered to BC screening at baseline (Table 1). The majority were non-smokers with a higher proportion among those in the adherent groups (54.51% vs. 62.94%, *p* < 0.001), around a quarter were ex-smokers (23.68% vs. 24.61%). More than 80% of women were drinkers (Low-risk drinkers 71.09% vs. 77.53% and risky/high-risk drinkers 6.78% vs. 4.90%), and half of the women had overweight/obesity in both groups (non-adherent 51.08% vs. adherent 53.38%). In addition to smoking status and alcohol consumption, the distribution across categories of education, country of birth, marital status, BC incidence, HRT use, and menopausal type also differed by BC screening adherence. Other individual characteristics were similar between non-adherent and adherent participants across all categories. Baseline characteristics were not significantly different between women diagnosed with BC and those not diagnosed with BC (Table S1).

**Table 1:** Baseline characteristics in 2001 by breast cancer screening adherence (*n* = 10,579).

Characteristics		Adherence to BC screening		*p*-value
		No (*n* = 2449)	Yes (*n* = 8130)	
Area of residence				
Major cities		35.52 (870)	33.91 (2757)	*p* = 0.013
Inner regional		41.36 (1013)	40.36 (3281)	
Outer regional		18.82 (461)	20.53 (1669)	
Remote/very remote		3.63 (89)	4.58 (372)	
Missing		0.65 (16)	0.63 (51)	
Education				
No education		61.98 (1518)	65.26 (5306)	*p* = 0.005
School-Diploma		20.25 (496)	19.69 (1601)	
University-Higher		16.95 (415)	14.27 (1160)	
Missing		0.82 (20)	0.77 (63)	
Country of birth				
Australia		74.85 (1833)	77.75 (6321)	*p* = 0.019
Other English speaking		14.33 (351)	13.06 (1062)	
Non-English		9.31 (228)	8.22 (668)	
Missing		1.51 (37)	0.97 (79)	
Marital status				
Partnered		75.17 (1841)	82.68 (6722)	*p* = 0.001
Non-partnered		23.89 (585)	17.01 (1383)	
Missing		0.94 (23)	0.31 (25)	
Ability to manage on income				
Impossible-difficult		42.92 (1051)	36.97 (3006)	*p* = 0.766
Not too bad-easy		55.66 (1363)	62.03 (5043)	
Missing		1.43 (35)	1.00 (81)	
BC incidence				
Yes		3.96 (97)	5.83 (474)	*p* = 0.004
No		96.04 (2352)	94.17 (7656)	
Smoking status				
Non-smoker		54.51 (1335)	62.94 (5117)	*p* < 0.001
Ex-smoker		23.68 (580)	24.61 (2001)	
Current smoker		21.19 (519)	12.12 (985)	
Missing		0.61 (15)	0.33 (27)	
Alcohol consumption				
Non-drinker/rarely drinks		14.13 (346)	11.51 (936)	*p* < 0.001
Low-risk drinkers		71.09 (1741)	77.53 (6303)	
Risky/high-risk drinkers		6.78 (166)	4.90 (398)	
Missing		8.00 (196)	6.06 (493)	
BMI				
Underweight/normal weight		42.06 (1030)	40.57 (3298)	*p* = 0.222
Overweight/obesity		51.08 (1251)	53.38 (4340)	
Missing		6.86 (168)	6.05 (492)	
OC use				
No		96.77 (2370)	97.53 (7929)	*p* = 0.045
Yes		2.86 (70)	2.34 (190)	
Missing		0.37 (9)	0.14 (11)	
HRT use				
No		78.97 (1934)	63.85 (5191)	*p* < 0.001
Yes		20.99 (514)	36.15 (2939)	
Missing		0.04 (1)		
Menopausal status				
No		69.33 (1698)	67.71 (5505)	*p* = 0.938
Yes		29.85 (731)	31.76 (2582)	
Missing		0.82 (20)	0.53 (43)	
Menopausal type				
Surgical		27.6 (676)	29.83 (2425)	*p* = 0.001
HRT use		9.68 (237)	18.67 (1518)	
OCP use		2.25 (55)	1.96 (159)	
Pre-menopausal		10.98 (269)	7.91 (643)	
Peri-menopausal		19.4 (475)	16.84 (1369)	
Post-menopausal		28.71 (703)	24.17 (1965)	
Missing		1.39 (34)	0.63 (51)	

The HRs and 95% CIs for lifestyle factors and screening adherence on BC incidence were presented in Table 2. There was a significant positive association between alcohol consumption and BC incidence; the hazards were higher for both low-risk drinkers and risky/high-risk drinkers (aHR [95% CI] 1.58 [1.36–1.85] and 1.32 [1.03–1.69], respectively). Overweight/obesity was positively associated with BC incidence (1.31 [1.18–1.46]). There was a significant positive association between HRT use and BC incidence (1.54 [1.37–1.73]). Adherence to BC screening was statistically significantly associated with BC incidence (1.68 [1.46–1.94]). The adjusted HR showed a positive association between BC incidence for smoking and OC use, but it is not statistically significant (Table 2).

**Table 2:** Hazard ratio (HR) and 95% confidence interval (95% CI) of lifestyle factors, and screening adherence.

Characteristics		uHR^c^ (95% CI)	aHR^d^ (95% CI)
Smoking status^a^			
Non-smoker		Ref	Ref
Ex-smoker		0.97 (0.87–1.08)	1.00 (0.90–1.11)
Current smoker		1.03 (0.88–1.21)	1.17 (0.99–1.38)
Alcohol consumption^b^			
Non-drinker/rarely drinks		Ref	Ref
Low-risk drinkers		1.58 (1.36–1.84)	1.58 (1.36–1.85)
Risky/high-risk drinkers		1.42 (1.12–1.81)	1.32 (1.03–1.69)
BMI^b^			
Underweight/Normal weight		Ref	Ref
Overweight/Obesity		1.21 (1.10–1.34)	1.31 (1.18–1.46)
HRT use^b^			
No		Ref	Ref
Yes		2.13 (1.91–2.39)	1.54 (1.37–1.73)
OC use^b^			
No		Ref	Ref
Yes		1.53 (0.69–3.41)	1.38 (0.62–3.09)
Screening adherence^b^			
Non-adherent		Ref	Ref
Adherent		1.72 (1.50–1.97)	1.68(1.46–1.94)

^a^Adjusted for income, area of residence, marital status, education, country of birth.

^b^Adjusted for age, income, area of residence, marital status, education, country of birth.

^c^uHR=unadjusted hazard ratio, ^d^aHR=adjusted hazard ratio.

In Table 3, the four-way decomposition of the total effect of lifestyle factors (BMI, alcohol consumption, OC use, and HRT use) on BC incidence, mediated via screening adherence, was presented. These lifestyle variables were selected a priori based on biological and epidemiological plausibility, the strength and consistency of existing evidence, and their relevance to the study aim. Three of the lifestyle factors, had significant total excess risk ratios (TERR) or overall risk ratios. BMI (TERR [95% CI]: 1.30 [1.17–1.44), alcohol consumption (1.55 [1.31–1.79]), and HRT use (1.57 [1.39–1.75]) all indicated positive relationships between these measures and BC incidence. There was a significant CDE for BMI (ERR [95% CI]: 0.365 [0.159 to 0.571]), alcohol consumption (0.435 [0.148 to 0.723]), and OC use (-0.688 [-0.783 to -0.592]), indicating a direct effect of these factors on BC incidence independent of screening adherence. The pure indirect effect (PIE), demonstrating the effect mediated through screening adherence, was significant for alcohol consumption (ERR [95% CI]: 0.018 [0.008 to 0.029]), and HRT use (0.029 [0.021 to 0.037]). The interaction components (INTref and INTmed) were generally not significant. The proportion attributable to the CDE was substantial for BMI (120.48%) and alcohol consumption (79.02%) (Table 3 and Fig. S1), while the proportion attributable to PIE was smaller but significant for alcohol consumption (3.32%) (Table 3 and Fig. S1) and HRT use (5.08%). The component of the total excess mean survival-time ratio due to CDE was significantly different from the component of the total excess mean survival-time ratio due to PIE (*p* < 0.01).

**Table 3:** Four-way decomposition of the mediation effect of screening adherence on the association between lifestyle factors and breast cancer incidence.

Component	ERR	95% CI	*p*-values	PA
Body mass index (BMI)				
Total	0.303	0.167 ; 0.439	0.000	100.00
CDE	0.365	0.159 ; 0.571	0.001	120.48
INTref	− 0.066	− 0.257 ; 0.125	0.497	− 21.87
INTmed	− 0.001	− 0.003 ; 0.001	0.512	− 0.23
PIE	0.005	0.001 ; 0.009	0.018	1.63
TERR	1.303	1.167 ; 1.439	0.000	
Alcohol consumption				
Total	0.551	0.310 ; 0.792	0.000	100.00
CDE	0.435	0.148 ; 0.723	0.003	79.02
INTref	0.093	− 0.170 ; 0.356	0.488	16.90
INTmed	0.004	− 0.008 ; 0.016	0.490	0.75
PIE	0.018	0.008 ; 0.029	0.001	3.32
TERR	1.551	1.310 ; 1.792	0.000	
Oral contraception				
Total	0.326	− 0.742 ; 1.393	0.550	100.00
CDE	− 0.688	− 0.783 ; -0.592	0.000	− 211.28
INTref	1.050	− 0.050 ; 2.150	0.061	322.66
INTmed	− 0.029	− 0.090 ; 0.033	0.361	− 8.77
PIE	− 0.008	− 0.025 ; 0.008	0.301	− 2.61
TERR	1.326	0.258 ; 2.393	0.015	
Hormone replacement therapy				
Total	0.570	0.389 ; 0.752	0.000	100.00
CDE	0.308	− 0.017 ; 0.634	0.064	54.03
INTref	0.215	− 0.086 ; 0.515	0.162	37.66
INTmed	0.018	− 0.007 ; 0.044	0.163	3.24
PIE	0.029	0.021 ; 0.037	0.000	5.08
TERR	1.570	1.389 ; 1.752	0.000	

ERR, Excess relative risk; CDE, control direct effect; INTref, Reference interaction; INTmed, Mediated interaction; PIE, Pure indirect effect; TERR, Total excess risk ratio/overall risk ratio; PA, Proportion attributable.  All models were adjusted for age, income, area of residence, marital status, highest level of education, and country of birth.

## Discussion

In this longitudinal cohort study, we found that lifestyle factors were directly associated with a higher risk of BC diagnosis, independent of any mediation through screening adherence. There were also smaller but statistically significant pure indirect effects of alcohol consumption and HRT use on BC incidence, indicating that screening adherence partially mediates the relationship between these lifestyle factors and BC incidence. These findings contribute to our understanding of the effects of screening adherence in BC prevention strategies.

There is substantial evidence that higher BMI is associated with an increased risk of BC, especially among postmenopausal women. After menopause, adipose tissue produces and circulates higher levels of estrogen, which is linked to a higher incidence of BC^18,19^. Obesity also promotes tumorigenesis through insulin resistance, hyperinsulinemia, and chronic inflammation^20,21^. In our study, we found a substantial significant direct effect of BMI on BC incidence after accounting for screening adherence as a mediator, confirming that adiposity influences BC risk primarily through inherent physiological pathways rather than through screening adherence.

A modest increase of BC risk due to alcohol consumption has been consistently reported in previous studies. Several systematic reviews and meta-analyses have found a dose-response relationship of 7–10% increase in risk of BC with each 10 g/day increase in alcohol consumption^22–24^. There are different mechanisms that explain increased BC risk from alcohol consumption, such as disruption of estrogen metabolism, increased circulating estrogen, and the production of carcinogenic metabolites^25,26^. Our finding that alcohol consumption has a significant direct effect on BC incidence is consistent with existing evidence.

We found important insights on the influence of HRT use on the BC incidence and its interaction with screening adherence. The significant positive association between HRT use and screening adherence suggests that women using HRT were more likely to engage in regular BC screening. This is consistent with previous findings that HRT users are more likely to engage in healthcare, including preventive screening^27,28^. We also found significant direct (without mediated via screening adherence) effects of HRT use as well as pure indirect effects on BC incidence. This indicates that the increased BC risk due to HRT use is mediated by increased screening adherence. These findings highlight the complex interplay between lifestyle factors, screening adherence, and BC incidence, and are consistent with prior evidence that enhanced screening among HRT users may contribute to higher detection rates of BC^29–32^.

As noted earlier, early detection through mammography screening reduces BC-specific mortality. To date, there is limited knowledge on whether screening adherence is on the causal pathways between lifestyle factors and BC incidence. Our work suggests that it mediates the association between two risk-related factors (alcohol consumption and HRT use) and BC incidence. We found a significant positive association between adherence to screening programs and BC incidence. However, screening adherence itself is not a risk factor for developing BC. This finding supports the notion that individuals who adhere to screening are more likely to have cancer detected often at earlier, more treatable stages, due to increased surveillance rather than increased risk^33^. Although smaller, significant pure indirect effects highlight the mediating role of BC screening on the relationship between lifestyle factors and BC. Notably, these findings underline the importance of interpreting screening-associated risk within the comprehensive framework of health-related behaviours and integrating these interrelated factors in the design of prevention strategies^6,34^. Non-significant reference and mediation interactions indicated no substantial effects on the overall risk profiles. Future research should focus on enhancing causal inference by incorporating high-quality longitudinal data with biomarkers and genetic information in the analysis of four-way decomposition for a more comprehensive understanding of the BC risks pathways.

This study has several strengths. We analysed a large longitudinal dataset incorporating time-varying exposures and mediators, and confirmed BC cases through cancer registry data, and applied a four-way decomposition approach for decomposing the total effect into direct and mediated effects. In addition, this method offers a distinct understanding of the underlying causal mechanisms compared to the traditional mediation approach. This study has some limitations as well. We did not include dietary patterns or physical activity in our analysis due to variations in methods between survey waves, and genetic predispositions are not available in the ALSWH. Thus, the possibility of potential residual confounding could not be completely ruled out. Bias due to measurement errors may be a possibility due to the self-reported lifestyle factors and screening participation. Selection bias could be present as participants with greater health concerns may participate more in screening programs and exhibit certain lifestyle behaviours. In addition, non-significant reference and mediation interaction could be due to insufficient measurement precision. Nevertheless, this study provides insights into the complex association between lifestyle factors and BC incidence, as well as the mediating effects of screening adherence.

## Conclusion

This study found that several lifestyle factors, including BMI, alcohol consumption, and HRT use, significantly influenced BC incidence, regardless of screening adherence in the pathway of the association. Nonetheless, there was a modest but significant indirect effect mediated via screening adherence for alcohol consumption and HRT use. These findings underscore the complex nature of BC risk and highlight the importance of considering both lifestyle factors and screening adherence in cancer prevention strategies.

## Methods

### Study design and setting

This study used data from the Australian Longitudinal Study on Women’s Health (ALSWH) and the linked Australian Cancer Database (ACD). Participants in the ALSWH were randomly selected from the Medicare Australia database using stratified random sampling, with oversampling in rural and remote areas to ensure sufficient sample size for statistical comparisons based on rurality. Data collection involved mailed questionnaires administered approximately every three years. The estimated response rate was 53% to 56% (*n* = 13,714) of women born 1946–1951 who were invited^35^. Retention rates have been high, ranging from 82% to 91%. Further details about the cohort have been published elsewhere^36,37^.

The ALSWH was conducted in accordance with the principles of the Declaration of Helsinki. The Human Research Ethics Committee of the University of Newcastle and the University of Queensland approved this study. The Australian Institute of Health and Welfare ethics committee approved the linkage of ALSWH survey data with the ACD. Written informed consent was obtained from all participants.

### Participants

Women from the ALSWH 1946–1951 birth cohort who completed the 2001 survey (wave 3) were included; they were aged 50–55 at the time, aligning with the minimum target age of 50 years for the free population mammogram program in Australia^38,39^. Follow-up was conducted using subsequent surveys (waves 4–9), with the final survey administered in 2019. We excluded: (1) Women who opted out of linking their survey data to administrative data, (2) Diagnosed with BC before the survey wave 3 in 2001 (*n* = 417) (see ‘Exposure variables’), and (3) Those who participated in only one of the surveys 3 to 9 (*n* = 230). After exclusions, we analysed 10,579 participants’ data in the current study.

### Exposure variables

The primary exposure variables consisted of individual lifestyle factors, including: BMI; dichotomised as underweight/normal weight (< 25 kg/m^2^) and overweight/obesity (≥ 25 kg/m²), since the prevalence of underweight and obesity was small; alcohol consumption (non-drinker, rarely drinks/low-risk drinker, and risky/high-risk drinker)^40^; smoking status (non-smoker, ex-smoker, current smoker); HRT use (yes/no); and OC use (yes/no). Two questions were used to collect information on smoking status in the ALSWH “How often do you currently smoke cigarettes or any tobacco products?” and “In your lifetime, would you have smoked at least 100 cigarettes (or equivalent)?”. Based on the responses, a woman is classified as an ex-smoker if she does not currently smoke but has smoked at least 100 cigarettes in her lifetime. Current smokers are those who report smoking (at any frequency) at the time of the survey and have smoked at least 100 cigarettes in their lifetime. These variables were measured from the ALSWH survey wave 3, which was administered in 2001. The follow-up measures were collected every three years until 2019 (waves 4–9).

### Outcome

BC incidence was the outcome of interest, which was ascertained from the ACD using ICD-O-3 code C50 during the follow-up period from 2001 to December 2019, the latest available data. The ACD contains near-complete national data on malignant tumors, excluding basal cell and squamous cell carcinomas of the skin^41^. All new primary BC cases were included, therefore, our definition encompasses all primary invasive BC cases regardless of stage at diagnosis. Cancer data for the ALSWH participants have been deterministically linked across all Australian states and territories from January 1982 onwards, with the most recent data available up to 2019. Further details regarding the linked ACD are documented elsewhere^42^.

### Mediator

BC screening adherence was considered as the mediator variable. A binary variable (adherent vs. non-adherent) was generated at each follow-up survey using the question “When did you last have a mammogram?”. Participants who had a mammogram within the past 2 years were classified as adherent^39^; otherwise, they were classified as non-adherent at that survey point.

### Confounders

Potential confounders measured at baseline in 2001 included age; area of residence^43^; country of birth^44^; educational qualification; marital status; menopausal type; and ability to manage on income (Table 1).

### Statistical analyses

Baseline characteristics from survey wave 3 (2001) were explored using frequency for categorical variables and mean for continuous variables and compared using the chi-square test and two-sample t-tests, respectively. Pairwise comparisons were performed for variables with significant overall chi-square tests and more than two categories (Table S2), but these results were not included in the main text to avoid over-interpretation from multiple testing. We have also reported summary statistics of participants’ characteristics by survey waves from wave 4 to wave 9 to show how the distribution changes during the follow-up period (Table S3).The dataset was designed in a counting process^45^ style to accommodate the time-varying nature of the exposures and mediator. Women who died, were lost to follow-up, or were not diagnosed with BC by the end of follow-up in 2019 were censored. Hence, the Cox proportional hazards regression model with time-varying covariates was used to determine the association between lifestyle factors, screening adherence and BC incidence. Unadjusted and adjusted hazard ratios (uHR, aHR) and corresponding 95% confidence intervals (CI) were estimated.

### Variable selection for four-way decomposition analysis

A range of behavioural and reproductive factors, including smoking and OC use, were initially examined. For the four-way decomposition analyses, variables were selected a priori based on the strength and consistency of existing evidence^10^ and their relevance to the study aim. OC use was retained, whereas smoking was excluded due to less consistent evidence linking it to BC risk. Selection was guided by prior evidence and biological plausibility rather than statistical significance, to avoid bias^46^.

We used four-way decomposition^16^ to decompose the overall effect of lifestyle factors on BC incidence, in the presence of screening adherence as a mediator. It produces four components: (1) controlled direct effect (CDE) measures the effect of the exposure in the absence of the mediator; (2) Reference interaction (INTref) measures the effect due to interaction but not mediation; (3) Mediated interaction (INTmed) measures the effect due to both mediation and interaction together, and the exposure could affect the mediator; and (4) Pure indirect effect (PIE) measures the effect of the mediator in the absence of the exposure. Two regression models were fitted: (1) a Cox proportional hazard model for the outcome (time to BC incidence) as a function of the exposure (lifestyle factors), the mediator (screening adherence), their interaction and confounders; (2) A logistic regression model for the mediator (screening adherence) as a function of the exposure (lifestyle factors) and confounders. Delta methods were used to calculate the variance-covariance matrix of the estimated components^47^. The relative contribution of each component was calculated by dividing the estimate of a component by the total excess relative risk (TERR). Excess relative risk (ERR=TERR-1) decomposed the total effect of exposure on outcome into four components in the presence of a mediator. We have presented the breakdown of the total effect due to lifestyle factors on the incidence of BC into four components in the presence of screening adherence (Figure S2). All models were adjusted for age, ability to manage on income, country of birth, area of residence, marital status, and highest level of education.

Missing values were imputed using data from the nearest adjacent surveys. There was less than 2% missing information for all variables except BMI and alcohol consumption (approximately 6%), and we treated these two variables as missing indicators in the modelling.

**Fig. 1:**
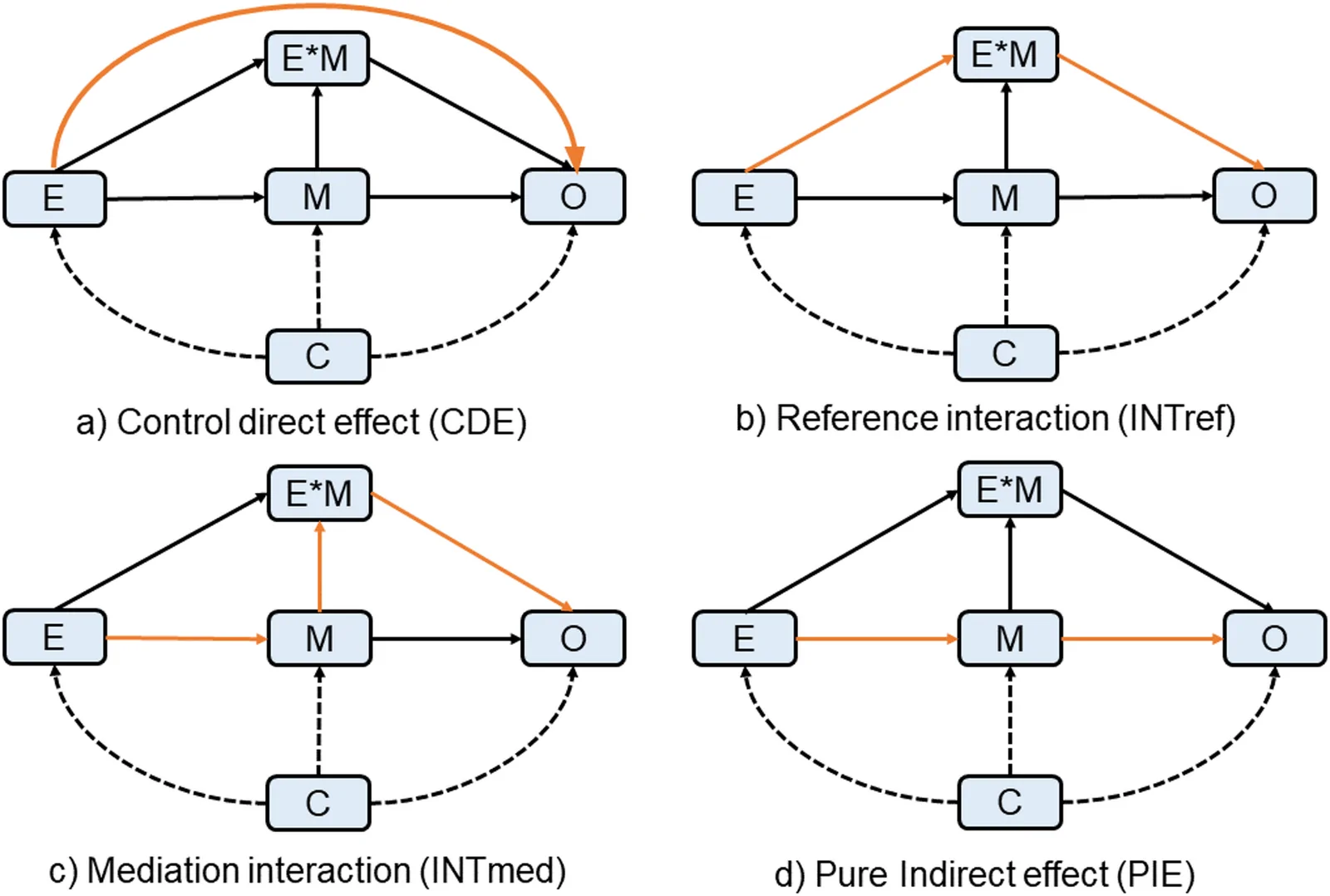
Four-way decompositions of the association between lifestyle factors on BC incidence, in the presence of screening adherence as a mediator. E: the exposures (BMI, alcohol consumption, HRT use, and OC use); M: the mediator (Screening adherence); E*M: the interaction between the exposure and the mediator; O: the outcome (time to the incidence of BC); C: a set of confounders (age, ability to manage on income, country of birth, area of residence, marital status, and highest level of education). Orange line shows each effect.

The CDE represents the direct influence of lifestyle factors on BC incidence (Fig. 1a). The INTref highlights the interaction between lifestyle factors and screening adherence on BC incidence, independent of the effect of lifestyle factors on screening adherence (Fig. 1b). This interaction characterises effects exclusively from the interaction itself, without mediation. The INTmed occurs only if lifestyle factors affect screening adherence, emphasizing a combined effect due to both mediation and interaction (Fig. 1c). Lastly, the PIE captures the effect of lifestyle factors on BC incidence only through its impact on screening adherence (Fig. 1d), specifying an effect simply from mediation without interaction. To minimize Type-I error due to large statistical power^48^, a more stringent level of significance (two-sided) was used at *P* < 0.01. All analyses were conducted using STATA version 18 (StataCorp LLC., College Station, TX, USA).

## Supplementary Information

Below is the link to the electronic supplementary material.

Supplementary Material 1

## Data Availability

As data custodian of ALSWH University of Newcastle and University of Queensland approved ethical application of this study (EoI #A1394: The influence of lifestyle factors on breast cancer incidence). Data were stored in the Secure Unified Research Environment facility, a remote access-computing environment to which authorized researchers were given encrypted access with strong authentication. The authors are not permitted to share any data.Data from the ALSWH are available to researchers on application. Information on the data access procedures, application forms and further details can be found at the ALSWH website (https://alswh.org.au/for-data-users/).
