# Supplementary material for: Association between risk-related lifestyle factors, screening adherence and breast cancer incidence: a decomposition analysis
Source: Sci Rep. 2026 Jun 22;16:28510. doi: 10.1038/s41598-026-58989-8 (PMC13572556; doi:10.1038/s41598-026-58989-8)
Supplement: Supplementary file 1 [file 41598_2026_58989_MOESM1_ESM.docx]

**Supplementary Materials**

Table S1: Baseline characteristics in 2001 by breast cancer incidence (N=10,579)

| Characteristics | Diagnosed with breast cancer | | p-value |
| --- | --- | --- | --- |
|  | No (N=10008) | Yes (N=571) |  |
| **Age** | 52.50 (1.46) | 52.53 (1.43) | 0.639 |
| **Smoking Status** |  |  |  |
| Non-smoker | 60.96 (6101) | 61.47 (351) | p=0.876 |
| Ex-smoker | 24.46 (2448) | 23.29 (133) |  |
| Current smoker | 14.18 (1419) | 14.89 (85) |  |
| Missing | 0.4 (40) | 0.35 (2) |  |
| **Alcohol consumption** |  |  |  |
| Non-drinker/rarely drinks | 12.28 (1229) | 9.28 (53) | p=0.256 |
| Low-risk drinkers | 75.93 (7599) | 77.93 (445) |  |
| Risky/high-risk drinkers | 5.32 (532) | 5.60 (32) |  |
| Missing | 6.47 (648) | 7.18 (41) |  |
| **BMI** |  |  |  |
| Underweight/Acceptable weight | 41.23 (4126) | 35.38 (202) | p=0.058 |
| Overweight/Obesity | 52.54 (5258) | 58.32 (333) |  |
| Missing | 6.24 (624) | 6.3 (36) |  |
| **OC use** |  |  |  |
| No | 97.34 (9742) | 97.55 (557) | p=0.946 |
| Yes | 2.46 (246) | 2.45 (14) |  |
| Missing | 0.2 (20) |  |  |
| **HRT use** |  |  |  |
| No | 67.54 (6759) | 64.1 (366) | p=0.443 |
| Yes | 32.45 (3248) | 35.9 (205) |  |
| Missing | 0.01 (1) |  |  |
| **Menopausal status** |  |  |  |
| No | 68.12 (6817) | 67.6 (386) | p=0.938 |
| Yes | 31.29 (3132) | 31.7 (181) |  |
| Missing | 0.59 (59) | 0.7 (4) |  |
| **Menopausal type** |  |  |  |
| Surgical | 29.39 (2941) | 28.02 (160) | p=0.064 |
| HRT use | 16.39 (1640) | 20.14 (115) |  |
| OCP use | 2.02 (202) | 2.1 (12) |  |
| Pre-menopausal | 8.43 (844) | 11.91 (68) |  |
| Peri-menopausal | 17.47 (1748) | 16.81 (96) |  |
| Post-menopausal | 25.48 (2550) | 20.67 (118) |  |
| Missing | 0.83 (83) | 0.35 (2) |  |
| **Education** |  |  |  |
| No education | 64.56 (6461) | 63.57 (363) | p=0.663 |
| School-Diploma | 19.75 (1977) | 21.02 (120) |  |
| University-Higher | 14.88 (1489) | 15.06 (86) |  |
| Missing | 0.81 (81) | 0.35 (2) |  |
| **Area of residence (city)** |  |  |  |
| Major cities | 34.4 (3443) | 32.22 (184) | p=0.935 |
| Inner regional | 40.49 (4052) | 42.38 (242) |  |
| Outer regional | 20.08 (2010) | 21.02 (120) |  |
| Remote/Very remote | 4.39 (439) | 3.85 (22) |  |
| Missing | 0.64 (64) | 0.53 (3) |  |
| **Country of birth** |  |  |  |
| Australia | 76.9 (7696) | 80.21 (458) | p=0.393 |
| Other English speaking | 13.45 (1346) | 11.73 (67) |  |
| Non-English | 8.53 (854) | 7.36 (42) |  |
| Missing | 1.12 (112) | 0.7 (4) |  |
| **Marital status** |  |  |  |
| Partnered | 80.88 (8094) | 82.14 (469) | p=0.900 |
| Non-partnered | 18.67 (1868) | 17.51 (100) |  |
| Missing | 0.46 (46) | 0.35 (2) |  |
| **Ability to manage on income** |  |  |  |
| Impossible-difficult | 38.33 (3836) | 38.7 (221) | p=0.766 |
| Not too bad-easy | 60.58 (6063) | 60.07 (343) |  |
| Missing | 1.09 (109) | 1.23 (7) |  |

Table S2: Pairwise comparison between groups of individual characteristic with more than two categories

| Characteristics | Pairwise comparisons | p-value |
| --- | --- | --- |
| Area of residence | Major cities vs inner regional | 0.913 |
|  | Major cities vs outer regional | 0.023 |
|  | Major cities vs remote/very remote | 0.018 |
|  | Inner regional vs outer regional | 0.041 |
|  | Inner regional vs remote/very remote | 0.031 |
|  | Outer regional vs remote/very remote | 0.964 |
| Education | No education vs School-Diploma | 0.374 |
|  | No education vs University-Higher | 0.027 |
|  | School-Diploma vs University-Higher | 0.140 |
| Country of birth | Australia vs other English speaking | 0.182 |
|  | Australia vs other non-English speaking | 0.140 |
|  | other English speaking vs other non-English speaking | 0.913 |
| Smoking status | Non-smoker vs ex-smoker | 0.322 |
|  | Non-smoker vs current smoker | <0.001 |
|  | Ex-smoker vs current smoker | <0.001 |
| Alcohol consumption | Non-drinker/rarely drinks vs low-risk drinkers | 0.224 |
|  | Non-drinker/rarely drinks vs risky/high-risk drinkers | 0.016 |
|  | low-risk drinkers vs risky/high-risk drinkers | 0.010 |
| Menopausal type | Surgical vs HRT use | <0.001 |
|  | Surgical vs OCP use | 0.544 |
|  | Surgical vs pre-menopausal | <0.001 |
|  | Surgical vs peri-menopausal | 0.155 |
|  | Surgical vs post-menopausal | 0.466 |
|  | HRT use vs OCP use | <0.001 |
|  | HRT use vs pre-menopausal | <0.001 |
|  | HRT use vs peri-menopausal | <0.001 |
|  | HRT vs post-menopausal | <0.001 |
|  | OCP use vs pre-menopausal | 0.001 |
|  | OCP use vs peri-menopausal | 0.934 |
|  | OCP use vs post-menopausal | 0.692 |
|  | Pre-menopausal vs peri-menopausal | <0.001 |
|  | Pre-menopausal use vs post-menopausal | <0.001 |
|  | Peri-menopausal vs post-menopausal | 0.361 |

Table S3: Characteristics of survey rounds used in the study of mid- aged cohort (1946-1951) of ALSWH

| Characteristics | Wave 4  (N=10184) | Wave 5  (N=9916) | Wave 6  (N=9317) | Wave 7  (N=8571) | Wave 8  (N=8129) | Wave 9  (N=7516) |
| --- | --- | --- | --- | --- | --- | --- |
|  | % (N) | % (N) | % (N) | % (N) | % (N) | % (N) |
| **BMI** |  |  |  |  |  |  |
| Underweight | 1.01 (103) | 1.08 (107) | 1.21 (113) | 1.12 (96) | 1.39 (113) | 1.37 (103) |
| Normal weight | 36.91 (3759) | 36.49 (3618) | 34.8 (3242) | 33.49 (2870) | 32.59 (2649) | 31.45 (2364) |
| Overweight | 31.31 (3189) | 32.9 (3262) | 33.57 (3128) | 32.6 (2794) | 31.82 (2587) | 29.51 (2218) |
| Obesity | 24.81 (2527) | 26.9 (2667) | 28.01 (2610) | 29.14 (2498) | 29.17 (2371) | 25.28 (1900) |
| Missing | 5.95 (606) | 2.64 (262) | 2.4 (224) | 3.65 (313) | 5.03 (409) | 12.39 (931) |
| **Physical Activity** |  |  |  |  |  |  |
| Nil/Sedentary | 15.01 (1529) | 14.68 (1456) | 15.36 (1431) | 16.38 (1404) | 16.04 (1304) | 16.52 (1242) |
| Low | 23.16 (2359) | 20.78 (2061) | 21.5 (2003) | 19.88 (1704) | 18.83 (1531) | 23.98 (1802) |
| Moderate | 22 (2240) | 22.2 (2201) | 20.45 (1905) | 20.56 (1762) | 20.33 (1653) | 20.93 (1573) |
| High | 33.16 (3377) | 36.35 (3604) | 36.21 (3374) | 39.59 (3393) | 40.62 (3302) | 34.18 (2569) |
| Missing | 6.67 (679) | 5.99 (594) | 6.48 (604) | 3.59 (308) | 4.17 (339) | 4.39 (330) |
| **Smoking** |  |  |  |  |  |  |
| Non-smoker | 59.16 (6025) | 59.93 (5943) | 60.86 (5670) | 62.41 (5349) | 62.66 (5094) | 64.12 (4819) |
| Ex. smoker | 27.13 (2763) | 28.83 (2859) | 30.06 (2801) | 30.17 (2586) | 31.09 (2527) | 30.39 (2284) |
| Current Smoker | 13.38 (1363) | 10.84 (1075) | 8.77 (817) | 6.57 (563) | 5.25 (427) | 4.16 (313) |
| Missing | 0.32 (33) | 0.39 (39) | 0.31 (29) | 0.85 (73) | 1 (81) | 1.33 (100) |
| **OC use** |  |  |  |  |  |  |
| No | 99.3 (10113) | 99.5 (9866) | 99.59 (9279) |  |  |  |
| Yes | 0.7 (71) | 0.14 (14) | 0.1 (9) | Not available | Not available | Not available |
| Missing |  | 0.36 (36) | 0.31 (29) |  |  |  |
| **HRT use** |  |  |  |  |  |  |
| No | 77.86 (7929) | 44.56 (4419) | 87.55 (8157) | 90.35 (7744) | 91.2 (7414) | 91.95 (6911) |
| Yes | 22.14 (2255) | 54.75 (5429) | 11.9 (1109) | 9.37 (803) | 8.54 (694) | 7.08 (532) |
| Missing |  | 0.69 (68) | 0.55 (51) | 0.28 (24) | 0.26 (21) | 0.97 (73) |
| **Alcohol consumption** |  |  |  |  |  |  |
| Low risk | 53.85 (5484) | 56.47 (5600) | 55.28 (5150) | 53.84 (4615) | 53.76 (4370) | 67.72 (5090) |
| Non-drinker | 14.94 (1521) | 13.75 (1363) | 14.22 (1325) | 16.15 (1384) | 16.95 (1378) | 7.74 (582) |
| Rarely drinks | 23.5 (2393) | 22.54 (2235) | 23.02 (2145) | 22.83 (1957) | 21.54 (1751) | 11.59 (871) |
| Risky drinkers | 5.71 (581) | 5.46 (541) | 6.03 (562) | 5.33 (457) | 5.43 (441) | 4.23 (318) |
| High risk drinkers | 0.93 (95) | 0.81 (80) | 0.65 (61) | 0.54 (46) | 0.6 (49) | 0.19 (14) |
| Missing | 1.08 (110) | 0.98 (97) | 0.79 (74) | 1.31 (112) | 1.72 (140) | 8.53 (641) |
| **Menopausal status** |  |  |  |  |  |  |
| Surgical | 31.31 (3189) | 33.09 (3281) | 33.59 (3130) |  |  |  |
| HRT | 9.82 (1000) | 6.47 (642) | 3.78 (352) |  |  |  |
| OCP | 0.47 (48) | 0.08 (8) |  |  |  |  |
| Pre | 2.04 (208) | 0.17 (17) | 0.01 (1) | Not available | Not available | Not available |
| Peri | 9.65 (983) | 1.99 (197) | 0.2 (19) |  |  |  |
| Post | 46.33 (4718) | 58.01 (5752) | 60.34 (5622) |  |  |  |
| Missing | 0.37 (38) | 0.19 (19) | 2.07 (193) |  |  |  |
| **Education** |  |  |  |  |  |  |
| No education/high school | 16.06 (1636) | 15.93 (1580) | 14.96 (1394) | 14.02 (1202) | 13.68 (1112) | 12.89 (969) |
| Trade/diploma | 68.21 (6946) | 68.21 (6764) | 68.75 (6405) | 68.78 (5895) | 68.79 (5592) | 68.79 (5170) |
| University or higher | 14.92 (1519) | 15.06 (1493) | 15.51 (1445) | 16.45 (1410) | 16.82 (1367) | 17.64 (1326) |
| Missing | 0.82 (83) | 0.8 (79) | 0.78 (73) | 0.75 (64) | 0.71 (58) | 0.68 (51) |
| **Area of residence** |  |  |  |  |  |  |
| Major cities | 37.49 (3818) | 37.3 (3699) | 37.51 (3495) | 38.19 (3273) | 38.73 (3148) | 38.89 (2923) |
| Inner regional | 39.17 (3989) | 39.25 (3892) | 40.29 (3754) | 39.8 (3411) | 40.46 (3289) | 41.18 (3095) |
| Outer regional | 19.4 (1976) | 19.43 (1927) | 18.62 (1735) | 18.84 (1615) | 17.85 (1451) | 17.14 (1288) |
| Remote | 3 (306) | 2.9 (288) | 2.64 (246) | 2.23 (191) | 2.04 (166) | 1.97 (148) |
| Very remote | 0.82 (83) | 0.84 (83) | 0.77 (72) | 0.63 (54) | 0.44 (36) | 0.39 (29) |
| Missing | 0.12 (12) | 0.27 (27) | 0.16 (15) | 0.32 (27) | 0.48 (39) | 0.44 (33) |
| **Marital Status** |  |  |  |  |  |  |
| Partnered | 79.46 (8092) | 77.81 (7716) | 76.29 (7108) | 73.97 (6340) | 71.36 (5801) | 67.62 (5082) |
| Non-partnered | 19.16 (1951) | 21.36 (2118) | 22.96 (2139) | 25 (2143) | 27.64 (2247) | 30.53 (2295) |
| Missing | 1.38 (141) | 0.83 (82) | 0.75 (70) | 1.03 (88) | 1 (81) | 1.85 (139) |

Figure S1. Breakdown of the total effect of alcohol drinking on breast cancer incidence into its four component parts due to mediation through screening adherence and additive interaction with screening adherence; dashed lines represent pure direct and total indirect effects which sum to the total effect.

 Figure S2. Breakdown of the total effect due to lifestyle factors on incidence of breast cancer, into the four component parts, (and their subsequent inter relationships) relating to the role of screening adherence.
